# Immunogenicity and Protection After Vaccination With a Modified Vaccinia Virus Ankara-Vectored Yellow Fever Vaccine in the Hamster Model

**DOI:** 10.3389/fimmu.2018.01756

**Published:** 2018-08-02

**Authors:** Justin G. Julander, Marco Testori, Cédric Cheminay, Ariane Volkmann

**Affiliations:** ^1^Institute for Antiviral Research, Utah State University, Logan, UT, United States; ^2^Bavarian Nordic GmbH, Martinsried, Germany

**Keywords:** yellow fever, 17D, passive immunization, hamster, modified vaccinia virus Ankara, neutralizing antibodies

## Abstract

The highly efficacious live-attenuated 17D yellow fever (YF) vaccine is occasionally associated with rare life-threatening adverse events. Modified vaccinia virus Ankara (MVA), a non-replicating poxvirus, has been used as a vaccine platform to safely deliver various antigens. A MVA-based YF vaccine (MVA-BN-YF) was tested with and without a non-mineral oil adjuvant in a hamster model of lethal YF disease and protective efficacy of this vaccine was compared with the 17D vaccine. The vaccine candidate MVA-BN-YF generated a protective response in hamsters infected with YFV that was comparable to protection by the live 17D vaccine. Similar levels of neutralizing antibody were observed in animals vaccinated with either vaccine alone or vaccine with adjuvant. Significant improvement in survival, weight change, and serum alanine aminotransferase levels were observed in vaccinated hamsters when administered 42 and 14 days prior to challenge with Jimenez YF virus (YFV). Neutralizing antibodies induced by MVA-BN-YF were transferred to naïve hamsters prior to virus challenge. Passive administration of neutralizing antibody 24 h prior to virus infection resulted in significantly improved survival and weight change. A trend toward reduced liver enzyme levels was also observed. MVA-BN-YF, therefore, represents a safe alternative to vaccination with live-attenuated YFV.

## Introduction

Yellow fever (YF), a hemorrhagic disease with jaundice, occurs throughout endemic areas of South America and Africa (1, 2). The etiologic agent, YF virus (YFV) is a mosquito-born flavivirus. The development of a live-attenuated vaccine in the early twentieth century significantly decreased the incidence of YF (3). The live-attenuated 17D vaccine, supplied by seven manufacturers, is currently used to protect travelers and also in childhood vaccination programs in many affected countries, with millions of doses distributed annually (4). Extensive use of this vaccine to combat a recent emergence event in Angola strained the supply of vaccine worldwide and demonstrated some limitation of availability and accessibility of the 17D vaccines (5), suggesting a need for an alternative vaccine. The virus also continues to emerge in new areas, including a 2017 outbreak in Brazil (6) and tens of millions of people could soon be at risk of an urban YF outbreak (7).

Serious adverse events, such as yellow fever vaccine-associated viscerotropic disease (YEL-AVD) and neurotropic disease (YEL-AND), have been reported after vaccination with the 17D vaccine. Incidences of YEL-AVD and YEL-AND in the United States are reported as 0.4 and 0.8 per 100,000 vaccinations, respectively (8). YEL-AVD can be quite severe with a case fatality rate of 65% (9). Moreover, incidence rates of adverse events are much higher in infants and the elderly (CDC Yellow Book, 2012). Despite the infrequency of adverse events, these considerations have stimulated efforts to develop safer YF vaccines.

The modified vaccinia virus Ankara (MVA) is a highly attenuated vaccinia virus strain that is non-replicating in humans. It has been used extensively as an antigen delivery vector. Moreover, MVA (MVA-BN^®^/IMVANEX^®^/IMVAMUNE^®^) has been licensed as safer smallpox vaccine in Europe and Canada. There is an extensive safety record of MVA in various vaccine platform applications (10–13). We have developed an MVA-vectored YF vaccine expressing the poly-protein PreM-E of YFV. In mice, the vaccine was shown to induce neutralizing antibodies (nAbs) that were increased by a non-mineral oil adjuvant (unpublished observation), but efficacy could not be tested in this model.

Challenge of hamsters with the adapted Jimenez strain of YFV causes viscerotropic disease after IP inoculation that is similar to disease in humans (14, 15). This model has been used in the evaluation of both antiviral interventions (16–19) and vaccines (20, 21). We have evaluated immunogenicity and efficacy of MVA-BN vectored YF and the approved live 17D vaccines in this model. Moreover, protective levels of nAb present after vaccination with MVA-BN YF were tested in naïve hamsters upon passive transfer. The results of these studies provide proof of principle for advancement of this investigational vaccine toward clinical trials.

## Materials and Methods

### Animals

Female golden hamsters (*Mesocricetus auratus)* with an average weight of 100 g were obtained from Charles River Laboratories (Wilmington, MA, USA). Following a 48-h quarantine and 5-day acclimation period, animals were randomly assigned to groups and individually marked with ear tags. All work with animals was performed in the Biosafety Level 3 area of the AAALAC-accredited Laboratory Animal Research Center at Utah State University (USU). Hamsters were cared for under an animal use protocol approved by the Institutional Animal Care and Use Committee Laboratory Animals (IACUC) at USU.

### Viruses

YF virus 17D was prepared by passaging in a monolayer culture of Vero cells and by harvesting cell culture fluid at the appearance of cytopathic effects (CPE). The virus was incubated overnight at 4°C followed by quantification by plaque assay in Vero76 cells grown in 12-well plates under methylcellulose overlay. After 5 days of incubation at 37°C and 5% CO_2_, plates were fixed and stained with 0.3% crystal violet-formaldehyde and plaques were counted.

The Jimenez strain (South American genotype I, isolated in Panama, 1974) was used for hamster challenge studies. The virus was adapted by serial passage in hamster liver, as described by Tesh and colleagues (15). A seed stock was prepared from livers of hamsters, removed 3 days after virus injection and homogenized in a 2× volume of sterile phosphate-buffered saline. This virus stock had a titer of 10^6.0^ 50% cell culture infectious doses (CCID_50_)/mL. Hamsters were challenged IP with 0.2 mL of a 10^−4^ dilution of virus stock (20 CCID_50_/animal).

### Vaccine

MVA-BN YF was prepared by inserting the coding region of preM and E that are based on the naturally occurring sequence of YFV (NCBI Accession No NC_002031) into the MVA-BN^®^ backbone. The virus was propagated in primary chicken embryo fibroblast cells in serum-free conditions. Montanide (Montanide™ ISA 720 VG manufactured by SEPPIC S.A., France) was used as a non-mineral oil adjuvant mixed with MVA-BN-YF to obtain a stable emulsion.

YF-VAX^®^ (Sanofi Pasteur, Swiftwater, PA, USA) 17D YFV was obtained as a lyophilized powder and was suspended in the manufacturer-supplied buffer. A 1:10 dilution of the vaccine was prepared and animals were vaccinated with > 1.0 × 10^4^ plaque forming units (pfu) 14 days prior to virus challenge.

### Neutralization Tests

Antibody levels in serum were quantified using the PRNT_50_ as previously described (21). Briefly, samples of test sera were heat-inactivated (56°C, 30 min), serial diluted (twofold), and mixed with an equal volume of YF 17D virus containing 50–70 pfu, incubated for 16–20 h at 2–8°C, and inoculated onto Vero76 monolayers grown in 12-well plates. Monolayers were covered with an overlay medium (0.85% methylcellulose in DMEM with 10% fetal bovine serum) after adsorption for 1 h at 37°C. Plates were fixed and stained with crystal violet-formaldehyde after 5 days incubation at 37°C. The endpoint was the highest dilution of serum inhibiting plaques by 50% or more when compared with virus controls.

### Serum Aminotransferase Assays

Serum was collected *via* ocular sinus bleed on 6 dpi. Alanine aminotransferase (ALT) (SGPT) reagent (Teco Diagnostics, Anaheim, CA, USA) was used, and the protocol was altered for use in 96-well plates as described previously (14). The aminotransferase concentrations were determined per manufacturer’s instructions.

### Infectious Cell Culture Assay

Test serum samples collected 4 dpi were serially diluted and added to Vero 76 cells. Ten days later, CPE was used to identify the endpoint of infection. Four replicates were used to calculate the CCID_50_/mL.

### Protective Efficacy of MVA-BN YF

Hamsters were randomly assigned to groups of 10–15 animals. Animals were immunized s.c. with MVA-BN YF ± adjuvant on −42 and −14 dpi or s.c. on −14 dpi with YF-VAX. A 10^−4^ dilution (10^2.0^ CCID_50_/mL) of the virus was prepared in minimal essential media. Hamsters were challenged on day 0 with Jimenez YFV. Serum was collected on −1, 4, and 6 dpi from all surviving hamsters for quantification of neutralizing antibody, serum virus, and ALT, respectively. Hamsters were observed at least twice daily for mortality, and weights were taken daily from 0 to 8 dpi to track weight change. Early euthanasia criteria included lying prone, lack of motility or non-responsiveness. Animals that were humanely euthanized were recorded as a mortality event on the following day, as opposed to animals naturally succumbed to viral disease between mortality checks, which were recorded on the day they were found.

### Passive Antibody Transfer

Serum samples collected during the vaccination study were used in passive immunization studies. Serum pools were diluted 1:10–1:100. A volume of 0.5 mL of each serum dilution was administered IP to groups of 10 hamsters 24 h prior to virus challenge. Serum was collected just prior to virus challenge to determine the level of nAb present in the serum at that time. Twenty-four hours after antibody treatment, animals were challenged with virus and followed for mortality, weight change, ALT levels, and viremia. A group treated with immune serum (IS) from animals vaccinated with MVA-BN YF was sham-infected and served as a toxicity control. An additional group of untreated and uninfected animals was included as normal controls. Serum was collected just prior to virus challenge to determine the nAb titer of each group 1 day after treatment. Serum samples were collected from the retro-orbital sinus 4 and 6 days after challenge for the assessment of virus titer and serum ALT levels, respectively.

### Statistical Methods

Treatment group comparisons for continuous variables (weight change, ALT, and viremia) were performed by one-way ANOVA with a Bonferroni multiple comparison post-test analysis comparing the antibody-treated groups to the placebo control group.

## Results

### Vaccination Studies

**Figure 1:**
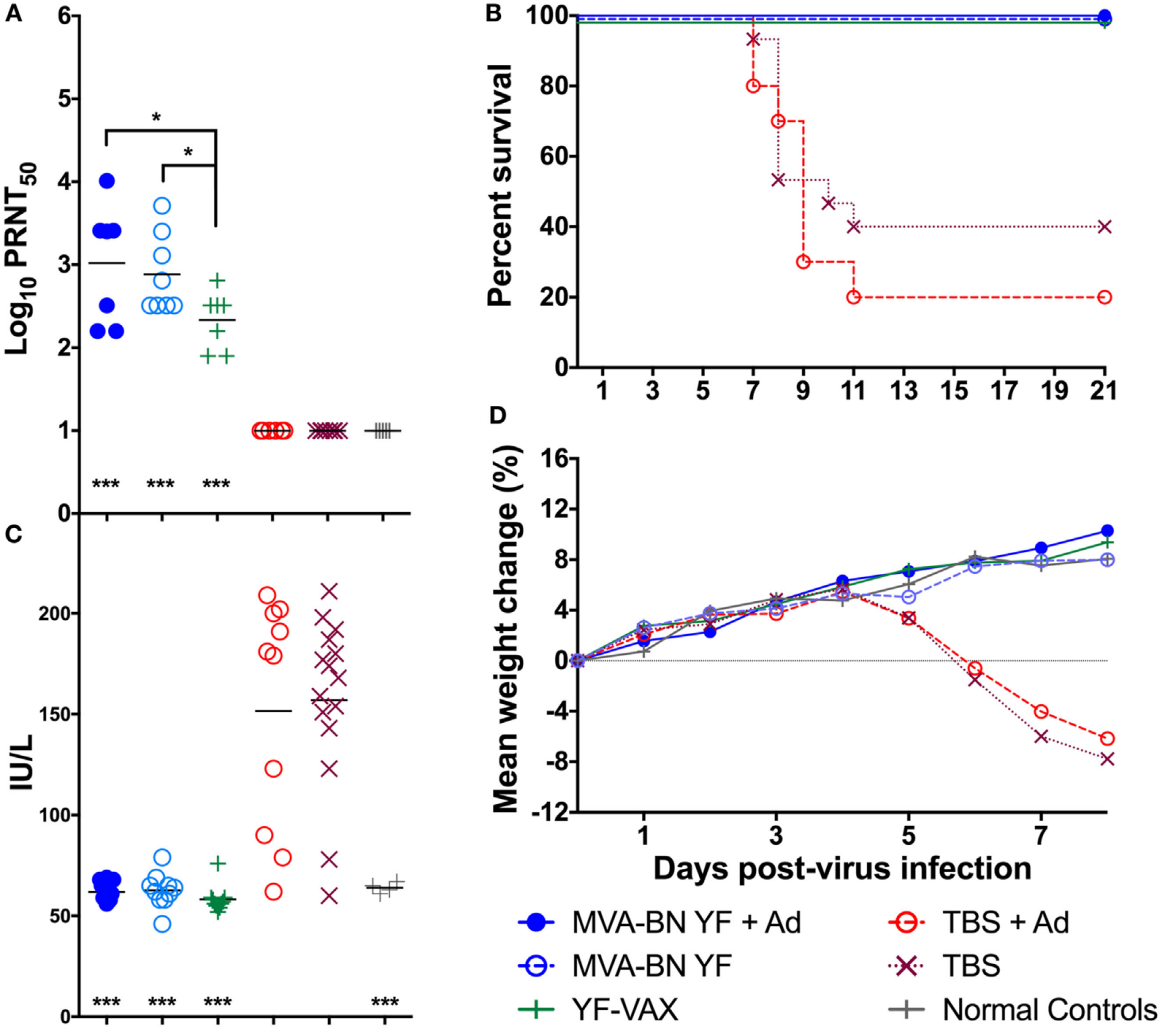
**(A)** Log_10_ plaque reduction neutralization titers to YF virus (YFV) were significantly elevated in hamsters vaccinated with MVA-BN YF ± adjuvant or with YF-VAX^®^ compared with animals vaccinated with vehicle [Tris-buffered saline (TBS)] ± adjuvant or untreated normal controls (*n* = 10/group). **(B)** Vaccination was protective in animals challenged with YFV and **(C)** serum levels of alanine aminotransferase on day 6 and **(D)** time-course weight change was improved in hamsters immunized with MVA-BN YF or YFVAX^®^ (***p < 0.001, when compared with vehicle).

Animals were vaccinated s.c. with 1 × 10^8^ TCID_50_ of MVA-BN-YF, with or without Montanide adjuvant, 42 and 14 days prior to challenge with YFV. The approved vaccine YF-VAX (Sanofi Pasteur) was used as a positive control and was administered s.c. 14 days prior to virus challenge. Daily monitoring of the animals after immunization revealed no observable adverse reaction to vaccination. In accordance with accepted correlates of protection, we quantified nAb titers in the serum of animals 24 h prior to virus challenge. Significantly (*p* < 0.001) elevated levels of nAb were observed in the serum of animals vaccinated with MVA-BN YF ± adjuvant or with YF-VAX when compared with Tris-buffered saline (TBS) ± adjuvant vaccinated controls or with normal controls (Figure 1A). There were also significantly (*p* < 0.05) higher levels of nAb in the serum of animals vaccinated with MVA-BN YF with and without adjuvant when compared with those from animals vaccinated with YF-VAX (Figure 1A). Unexpectedly, an adjuvant effect such as seen in mice (data not shown) was not detected (Figure 1A). The remainder of serum collected prior to virus challenge was pooled by treatment group and used in the subsequent passive Ab transfer study described below.

Vaccination with MVA-BN-YF ± adjuvant administered −42 and −14 days prior to virus challenge resulted in significant (*p* < 0.001) protection through the end of the study (Figure 1B; Table 1). In fact, complete survival was observed in all vaccinated animals, including those vaccinated with YF-VAX, when compared with 40 and 20% survival of vehicle control groups that were injected at the same time as vaccine with TBS or TBS + adjuvant as vehicle control, respectively (Figure 1B; Table 1).

**Table 1:** Efficacy of vaccination with MVA-BN YF on disease in hamsters challenged with YF virus.

		Toxicity controls			Infected, treated			
			
Treatment	Treatment dose, schedule	Alive/total	Serum alanine aminotransferase (ALT)^a^ (IU/L) ± SD	Mean wt. change^b^ (g) ± SD	Alive/total	MDD^c^ ± SD	Serum ALT^a^ (IU/L) ± SD	Mean wt. change^b^ (g) ± SD
MVA-BN YF	1 × 10^8^ TCID_50_, −42, −14 dpi	–	–	–	10/10	>21.0 ± 0.0	61.9 ± 5.0	4.8 ± 4.2
MVA-BN YF + Adj.	1 × 10^8^ TCID_50_, −42, −14 dpi	3/3	62.3 ± 9.1	5.0 ± 2.0	10/10	>21.0 ± 0.0	62.8 ± 8.3	4.6 ± 1.9
YF-VAX	>5.5 × 10^4^ pfu, −14 dpi	3/3	66.4 ± 18.0	4.3 ± 3.1	10/10	>21.0 ± 0.0	58.4 ± 6.6	4.6 ± 2.5
Tris-buffered saline (TBS) + Adj.	−42, −14 dpi	3/3	57.6 ± 5.9	−0.3 ± 6.1	2/10	8.6 ± 1.3	151.6 ± 57.0	−6.2 ± 7.3
TBS	−42, −14 dpi	3/3	62.5 ± 5.8	2.3 ± 1.2	6/15	8.4 ± 1.2	156.9 ± 42.3	−8.9 ± 7.1
Norm. controls	–	3/3	64.3 ± 2.6	5.0 ± 0.0	–	–	–	–

*^a^Serum ALT levels collected on 6 dpi*.

*^b^Difference between weight on 3 and 6 days post-virus challenge representing maximal weight change within this study*.

*^c^Mean day to death of animals that succumb to disease during the experimental period of 21 days*.

Serum ALT was significantly reduced to baseline levels in all animals vaccinated with MVA-BN YF or YF-VAX when compared with respective controls that showed elevated levels of the liver enzyme, a typical read-out for YFV infection (Figure 1C; Table 1). Animals vaccinated with MVA-BN YF ± adjuvant or with YF-VAX had a similar weight gain to uninfected control animals (Figure 1D). Vehicle-vaccinated animals infected with YFV experienced weight loss beginning 5 dpi, which continued to mortality and is similar to previous results in this model. Some weight loss was observed on day 5 in sham-infected animals treated with TBS + adjuvant, but this group gained weight after that time. Significant improvement in weight gain between 3 and 6 dpi was observed in hamsters immunized with MVA-BN YF ± adjuvant or with YF-VAX (Table 1).

### Passive Neutralizing Antibody Protection Studies

Naïve hamsters were treated i.p. with 1:10 or 1:100 dilutions of pooled IS collected on day 49, either from animals immunized with two injections (day 0 and 28) of MVA-BN YF emulsified in Montanide adjuvant, or from animals receiving one injection of YF-VAX (day 28) (Table 2). One day after IS transfer, animals were challenged with YFV. A serum sample was taken from each animal 4 h prior to virus challenge to determine the neutralizing Ab present in the serum at the time of infection.

**Table 2:** The effect of passively transferred immune serum (IS) from animals vaccinated with MVA-BN YF vaccine or with YF-VAX administered 24 h prior to challenge with YF virus.

			Toxicity controls			Infected, treated			
				
Treatment	Dose	Serum NeutAb titer^a^	Alive/total	Serum alanine aminotransferase (ALT)^b^ (IU/L) ± SD	Mean wt. change^c^ (g) ± SD	Alive/total	MDD^d^ ± SD	Serum ALT^b^ (IU/L) ± SD	Mean wt. change^c^ (g) ± SD
MVA-BN YF IS	1:10	1.1 ± 0.2	3/3	67 ± 9.0	4.7 ± 2.1	8/10***	10.0 ± 0.0	88 ± 34	5.3 ± 3.4**
MVA-BN YF IS	1:100	<1.0 ± 0.0	–	–	–	4/10	8.5 ± 1.0	206 ± 82	−7.5 ± 5.1
YF-VAX IS	1:10	1.0 ± 0.1	3/3	60 ± 6.0	6.0 ± 1.0	3/10	9.0 ± 1.5	162 ± 92	−4.9 ± 5.3
YF-VAX IS	1:100	1.0 ± 0.1	–	–	–	1/10	8.4 ± 0.9	170 ± 89	−7.5 ± 6.9
Placebo IS	1:10	<1.0 ± 0.0	3/3	73 ± 19	6.0 ± 3.0	1/15	9.3 ± 1.5	169 ± 99	−4.9 ± 8.3
Norm. controls	–		3/3	56 ± 6.0	4.7 ± 2.1	–	–	–	–

*^a^Average log_10_ neutralizing antibody titer of serum collected 4 h prior to virus challenge and quantified by PRNT_50_*.

*^b^Serum ALT levels collected on 6 dpi*.

*^c^Difference between weight on 3 and 6 days post-virus challenge representing maximal weight change within this study*.

*^d^Mean day to death of animals that succumb to disease during the experimental period of 21 days*.

***p < 0.001, **p < 0.01, when compared with placebo treatment.

**Figure 2:**
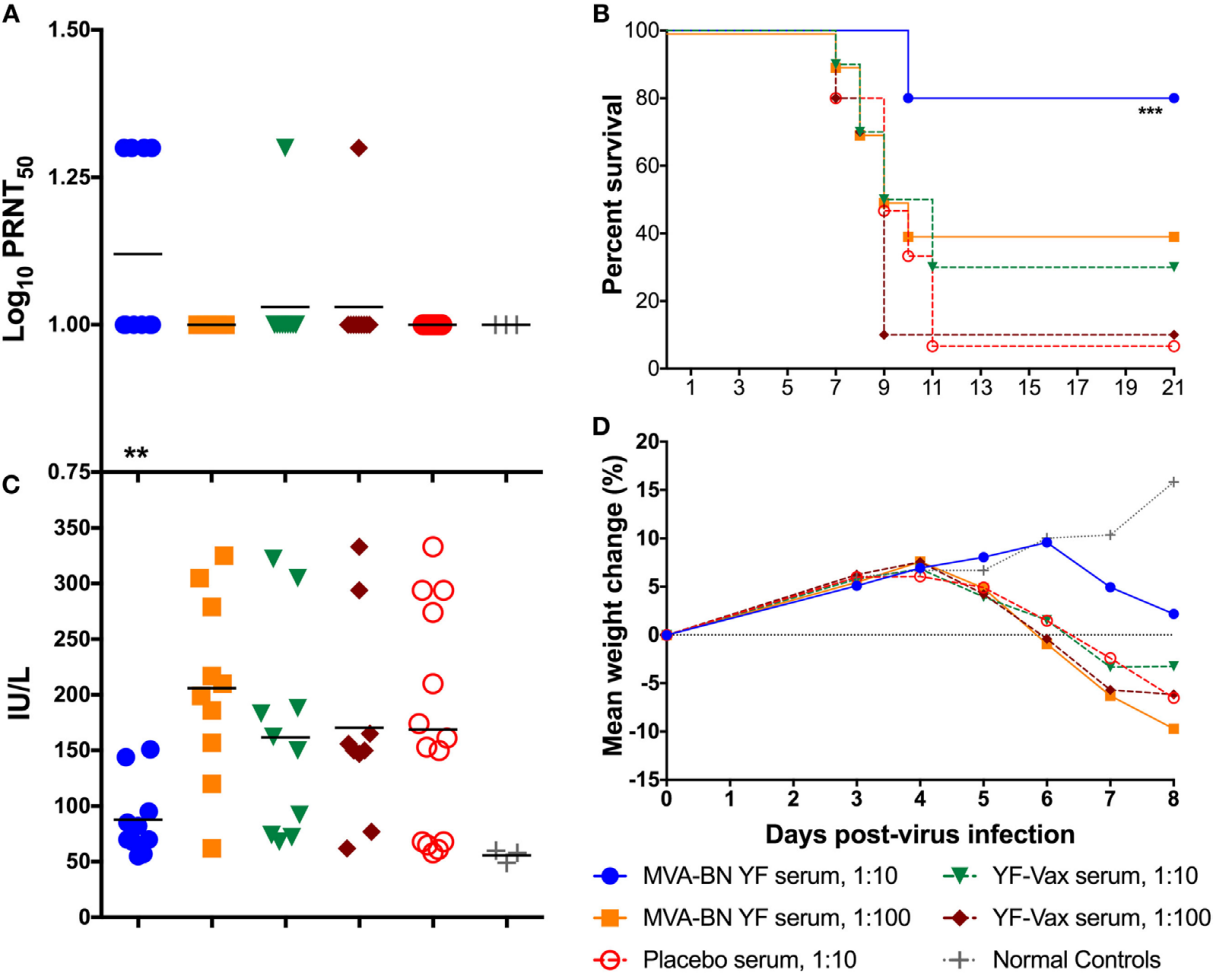
**(A)** Passive administration of serum from hamsters vaccinated with MVA-BN YF or YF-VAX^®^ resulted in low levels of neutralizing Ab in naïve hamsters (*n* = 10/group) present 24 h after treatment. **(B)** Survival of hamsters after passive administration of serum from vaccinated hamsters. Passive transfer was only significantly protective in hamsters receiving a 1:10 dilution of serum from hamsters vaccinated with MVA-BN YF. **(C)** Serum alanine aminotransferase on 6 days post challenge for individual hamsters passively immunized with various dilutions of immune serum generated after immunization with MVA-BN YF or YF-VAX^®^. **(D)** Time-course weight change from 0 to 8 dpi.

All of the animals treated with a 1:10 dilution of IS from the MVA-BN YF group had detectable (≥1 log_10_ PRNT_50_) neutralizing Ab titers present in the serum 24 h after treatment (Figure 2A; Table 3). The average PRNT_50_ for animals treated with 1:10 diluted MVA-BN YF pooled serum was significantly (*p* < 0.01) higher than that of animals treated with IS from placebo-vaccinated animals (Figure 2A; Table 2). Animals treated with a 1:100 dilution of IS from MVA-BN YF-vaccinated hamsters had PRNT_50_ levels at or below the level of detection (Table 2).

**Table 3:** Neutralizing antibody titers present in serum 24 h after treatment with a 1:10 dilution of immune serum.

		Log_10_ PRNT_50_^a^ (survival-y/n^b^)		
		
	Treatment	1:10 MVA-BN YF	1:10 YF-VAX	1:10 Placebo
Animal number	1	1.3 (y)	1.0 (y)	<1.0 (n)
	2	1.0 (y)	1.3 (n)	1.0 (n)
	3	1.0 (n)	1.0 (n)	<1.0 (n)
	4	1.3 (y)	1.0 (n)	<1.0 (n)
	5	1.0 (n)	1.0 (n)	<1.0 (n)
	6	1.3 (y)	1.0 (n)	<1.0 (n)
	7	1.0 (y)	1.0 (n)	<1.0 (y)
	8	1.0 (y)	<1.0 (y)	<1.0 (n)
	9	1.0 (y)	1.0 (y)	<1.0 (n)
	10	1.3 (y)	1.0 (n)	<1.0 (n)
	11			<1.0 (n)
	12			1.0 (n)
	13			1.0 (n)
	14			1.0 (n)
	15			<1.0 (n)

*The eventual survival (y) or mortality (n) through 21 dpi is indicated in parentheses*.

*^a^The log_10_ 50% of control*.

^b^Did the animal survive (yes or no)?

All but one animal from the 1:10 dilution of serum from animals vaccinated with YF-VAX had titers above the level of detection (≥1 log_10_ PRNT_50_), but the average was lower than that of the MVA-BN YF serum and was not significantly different from placebo treatment (Figure 2A; Table 3). All of the animals in the group treated with the 1:100 dilution of YF-VAX serum or with a 1:10 dilution of serum from placebo-vaccinated animals had neutralizing Ab titers at or below the limit of detection (Table 2). Neutralizing Ab titers corresponded well with predicted titers (Table 2). Higher levels of detectable neutralizing Ab also corresponded with increased protection, e.g., in the group treated with 1:10 dilution of MVA-BN YF serum, further demonstrating the protective effect of MVA-BN YF vaccination through neutralizing Ab.

Indeed, treatment with the 1:10 dilution of the serum from the group vaccinated with MVA-BN YF provided significant (*p* < 0.001) protection to naïve hamsters challenged with YFV as compared with placebo (Figure 2B). No significant protection was observed after passive administration of a 1:10 dilution of serum from animals immunized with YF-VAX, despite a higher survival rate when compared with placebo (Figure 2B).

Hamsters treated with the 1:10 dilution of MVA-BN YF IS also had lower serum ALT titer than all other groups, although this difference was not significant (*p* > 0.05) when compared with levels in animals treated with serum from placebo-vaccinated animals (Figure 2C). Typically, ALT and mortality are generally correlative in this model (14), but in this study, ALT levels in animals treated with IS from placebo-vaccinated animals were widely distributed and diminished statistical power for this parameter.

Improved weight change after 4 dpi was observed in hamsters in the group receiving the 1:10 dilution of serum from MVA-BN YF immunized animals (Figure 2D). This weight gain was similar to that of sham-infected groups. Indeed, the difference in weight between 3 and 6 dpi was significantly (*p* < 0.01) improved in animals treated with 1:10 MVA-BN YF serum group when compared with infected hamsters treated with IS from placebo-vaccinated groups (Table 2). All other groups had weight loss similar to that of animals treated with serum from vehicle-vaccinated animals.

## Discussion

The investigational YFV vaccine MVA-BN YF provided protection in a hamster model of disease, as indicated by significant improvement in survival, weight change, and liver enzyme levels in the blood when compared with placebo vaccination. Protection corresponded with induced neutralizing Ab titers, which was demonstrated by passive transfer studies. Although millions of doses of the currently approved 17D-based vaccine have been used to protect from YFV infection and disease, a recent outbreak in Angola demonstrated the limitation of the global stockpile of this vaccine and underscores the potential need for the development of a safe and effective vaccine. Use of MVA-BN YF would improve some aspects of the current YFV vaccine, including reducing or eliminating rare adverse events associated with current vaccines (22). Although MVA-BN YF utilizes a live-attenuated virus, this replication deficient virus demonstrated a high level of safety and immunogenicity in around 7,000 vaccinated individuals (23), even in fully immune compromised mice and primates (24–26). The results reported herein suggest further testing toward clinical development is warranted.

The accepted immune correlate of protection against YF is neutralizing antibodies (27). A lethal primate challenge model of YF was used to assess the minimal protective level of neutralizing Ab elicited by the 17D YF vaccine, which was determined to be ≥0.7 log_10_ neutralization index of ≥0.7 (28). Natural infection or effective vaccination will elicit the production of neutralizing antibody, which will persist in the serum, protecting from subsequent challenge with the same virus or serotype. We have also demonstrated protection after immunization with an investigational vaccine in a hamster model of YFV infection and demonstrated neutralizing antibody as a correlate of protection (20, 21). Significantly elevated levels of neutralizing antibody were observed in hamsters vaccinated with MVA-BN YF, which were protective in passive administration studies. Typically, MVA-BN-based vaccines provide transient and localized expression of the gene of interest in animal models. We anticipate that preM and E expression will be inactive 2 weeks after vaccination with MVA-BN YF. Protection observed in these studies would be due to elicited immune responses as a result of vaccination.

Vaccination with YF-VAX was included as a control, although the different nature of YF-VAX and MVA-BN YF precluded a direct comparison. The currently approved YF-VAX is a live-attenuated vaccine that confers protection after a single dose, while MVA-BN YF is replication incompetent and has a relatively short period of antigen expression and would likely require a booster vaccination in a clinical setting. The potential disadvantage of a required booster vaccination is, in our view, compensated by a very good safety profile of the non-replicative MVA-BN YF and precludes the adverse events that are associated with YF-VAX. Previous studies demonstrated a greater degree of stimulation of neutralizing Ab production after immunization with YF-VAX, but a greater vaccine dose (>4.7 log_10_ pfu) was used in these studies (20, 21). The predicted protective value of a PRNT_50_ value of 40 (1.6 log_10_ PRNT_50_) was also higher than what was observed in this study, but this is likely well above functional protective levels and other studies indicate that Ab levels >1.0 log_10_ PRNT_50_ are protective in vaccinee (29). The addition of the non-mineral oil Montanide to MVA-BN YF did not increase antibody titers compared to the non-adjuvanted vaccine. This was in contrast to previous unpublished findings in BALB/c mice. However, differences in the mouse were greater after a single vaccination and the hamster studies reported here used a prime-boost vaccination schedule only.

Taken together, vaccination of hamsters with an MVA-based YF vaccine provided full protection that was comparable to the licensed 17D YF vaccine, and this protection could be transferred to naïve hamsters through serum, confirming that induced neutralizing antibodies are a correlate of protection. MVA-BN YF may represent a safe alternative to 17D YF vaccines.

## Ethics Statement

This study, including veterinary care and experimental procedures, was conducted in accordance with the approval of the Institutional Animal Care and Use Committee of Utah State University (USU) under the approved protocols #1231 and 2248. The work was performed in the AAALAC-accredited Laboratory Animal Research Center at Utah State University.

## Author Contributions

JJ designed and oversaw animal studies, interpreted the data, and wrote the manuscript. MT helped characterize the candidate vaccine and provided input on the manuscript. CC helped design and characterize the candidate vaccine and helped write the manuscript. AV helped design the studies, oversaw the development and characterization of the vaccine, and helped write the manuscript.

## Conflict of Interest Statement

The authors declare that the research was conducted in the absence of any commercial or financial relationships that could be construed as a potential conflict of interest.
